# The effect of lidocaine in breast cancer: a concise overview of preclinical and clinical evidence

**DOI:** 10.3389/fphar.2026.1750375

**Published:** 2026-03-20

**Authors:** Tao Gong, Yanan Zhao, Zixuan Wang, Yuqing Li, Pengjia Wang, Xuanzhi Feng, Peiyun Gao, Xuesen Su, Shouyuan Tian

**Affiliations:** 1 Anesthesiology College of Shanxi Medical University, Taiyuan, Shanxi, China; 2 The Department of Anesthesiology, The Fifth Clinical Medical College of Shanxi Medical University, Taiyuan, Shanxi, China; 3 Shanxi Key Laboratory of Geriatric Precision Anesthesia and Complication Prevention, The Fifth Clinical Medical College of Shanxi Medical University, Taiyuan, Shanxi, China; 4 Academy of Medical Sciences, Shanxi Medical University, Taiyuan, Shanxi, China

**Keywords:** anticancer mechanisms, breast cancer, clinical translation, drug combination, drug repurposing potential, lidocaine

## Abstract

Postoperative recurrence, distant metastasis, and chemotherapy resistance in breast cancer present significant challenges in clinical treatment. Improving perioperative intervention strategies is crucial for enhancing patient prognosis. Lidocaine, a commonly used local anesthetic, has recently been shown to possess potential anti-breast cancer effects, offering a novel therapeutic direction through its drug repurposing potential. This review explores the multifaceted mechanisms by which lidocaine exerts anti-cancer effects, including nuclear regulation, membrane receptor channel inhibition, tumor microenvironment reprogramming, autophagy activation, and synergistic effects when combined with chemotherapy agents. Furthermore, the review discusses the limitations of current research, incorporating clinical evidence. This article provides a theoretical foundation for the perioperative application of lidocaine in breast cancer and the development of combination therapies, while also outlining future research directions to promote the clinical translation of “old drugs, new uses.”

## Highlights

- Lidocaine exerts multiple anti–breast cancer effects beyond anesthesia, providing mechanistic evidence for its perioperative anticancer potential.
- It inhibits Cdc20-mediated mitotic progression, leading to G2/M phase arrest.
- Lidocaine regulates miRNA expression and DNA methylation, thereby inducing apoptosis and ferroptosis.
- It modulates receptor channels (TRPM7, TRPV6, and CXCR4) and the tumor microenvironment.

## 1 Introduction

Breast cancer accounts for 11.7% of new cancer cases in women globally, with approximately 685,000 deaths in 2020, making it the second leading cause of cancer-related death among women (Sung et al., 2021). Despite ongoing advancements in surgical resection, chemotherapy, and targeted therapies, the 5-year recurrence rate for breast cancer patients remains as high as 20%–30%, with distant metastasis (e.g., lung, bone, and brain metastases) being the leading cause of mortality (Martinez et al., 2015).

Lidocaine is an amide-type local anesthetic that exerts its pharmacological effects primarily through reversible blockade of voltage-gated sodium channels in neuronal and cardiac cell membranes, thereby inhibiting the initiation and propagation of action potentials. In the peripheral nervous system, this action produces local anesthesia and analgesia, while in myocardial tissue it reduces excitability and abnormal automaticity, effects that have supported its use in selected cardiac conditions (Butterworth and Strichartz, 1990). Clinically, lidocaine is widely used for infiltration anesthesia, peripheral nerve blocks, epidural anesthesia, and topical anesthesia, and it may also be administered intravenously in specific clinical settings (Harrison et al., 1977). Pharmacokinetic studies indicate that lidocaine has a rapid onset of action and undergoes extensive hepatic metabolism via the cytochrome P450 enzyme system, with a reported systemic elimination half-life of 1.5–2 h, which may be prolonged in patients with hepatic impairment or reduced cardiac output (Harrison et al., 1977; Rosenberg et al., 2004). Adverse effects predominantly involve the central nervous and cardiovascular systems, including dizziness, somnolence, sensory disturbances, tremor or seizures, as well as hypotension, bradycardia, and conduction disturbances; at elevated plasma concentrations, pronounced neurotoxicity and cardiotoxicity may occur, necessitating careful dose control and clinical monitoring (Butterworth and Strichartz, 1990; Rosenberg et al., 2004).

Despite its traditional application as a local anesthetic, a growing body of evidence suggests that lidocaine possesses pleiotropic biological activities that extend far beyond simple sodium channel blockade. In light of the high recurrence rates and therapeutic challenges in breast cancer management, the drug repurposing potential of lidocaine offers a promising new direction for breast cancer treatment. Preclinical studies have shown that lidocaine can inhibit breast cancer cell proliferation, induce apoptosis, and reduce metastatic potential. However, these findings still face considerable limitations regarding clinical translation. This review systematically elaborates on the core mechanisms by which lidocaine exerts its anti-breast cancer effects (Table 1) and analyzes clinical data on perioperative lidocaine applications. Furthermore, it provides theoretical support for the development of lidocaine-based breast cancer intervention strategies. The article aims to lay the foundation for promoting the clinical translation of lidocaine’s repurposing potential.

**TABLE 1:** Summary of lidocaine’s mechanisms and effects in breast cancer cells.

Cell line	Target	Key findings	Biological effect	References
MDA-MB-231	CDC 20	Induces G2/M cell cycle arrest	Cell viability↓	Das et al. (2023)
MCF 10A	CDC 20	-	No effect	Das et al. (2023)
T47D	miRNA	miR-382-5p/SLC7A11 Axis	Ferroptosis↑	Sun et al. (2021)
MCF-7	miRNA	miR-495-3p/FGF9 axis	Proliferation, Migration and Invasion↓	Lin et al. (2021)
BT-20 and MCF-7	DNA	Time- and dose-dependent demethylation	Apoptosis↑	Lirk et al. (2012), Lirk et al. (2014)
MCF-7,MDA-MB-231	DNA (CpG)	Global demethylation of tumor suppressors	Apoptosis↑	Li et al. (2014)
MDA-MB-231,AU565,T47D and MDA-MB-468	TRPM7	Inhibition of TRPM7 function	Viability & migration ↓	Liu H et al. (2021)
MDA-MB-231	TRPV6	Downregulation of TRPV6 expression and reduced Ca^2+^ influx rate	Migration ↓	Jiang et al. (2016)
MCF-7	VDAC 1	Downregulation of Bcl-2 and increased P53 expression	Apoptosis↑	Long et al. (2022)
MDA-MB-231	CXCR4	Inhibition of CXCL12 signaling, cytoskeletal remodeling suppressed	Metastasis ↓	D’Agostino et al. (2018)
MCF10A and MCF10A-Bcl2	McTNs	Inhibition of microtentacle extension	Metastasis ↓	Yoon et al. (2011)
4T1	Src pathway	Inhibition of MMP-2 and MMP-9 expression	Metastasis ↓	Wall T et al. (2019)
MDA - MB 231 and 4T1	TAMs/TGF-β	Reprogramming M2-TAMs to M1-type	Recurrence ↓	Seok Han et al. (2024)
MDA-MB231and 4T1	Nnat,NGFs	Suppression of neurofiber formation	Metastasis ↓	Li et al. (2021)
MDA-MB-231	Autophagy	Induction of autophagolysosome formation	Growth↓; Apoptosis↑	Chen et al. (2022)
BT-474	Autophagy	No significant effect on autophagy	No effect	Chen et al. (2022)
MCF-7 and MCF-10A	Caspase	Activation of caspases 7, 8, 9; PARP cleavage	Apoptosis↑	Chang et al. (2014)
MDA-MB-231	Drug combination	PI3K/AKT/GSK3β	Growth ↓; Apoptosis↑	Han et al. (2022)
MDA-MB-231	Drug combination	Lidocaine pathway inhibition	Metastasis↓; Apoptosis↑	Gao et al. (2018)
MCF-7	Drug combination	Increased reactive oxygen species (ROS), mitochondrial membrane damage	Apoptosis↑	Tabrizi and Chiniforoshan (2016)

## 2 Nuclear-associated regulatory mechanisms

**FIGURE 1:**
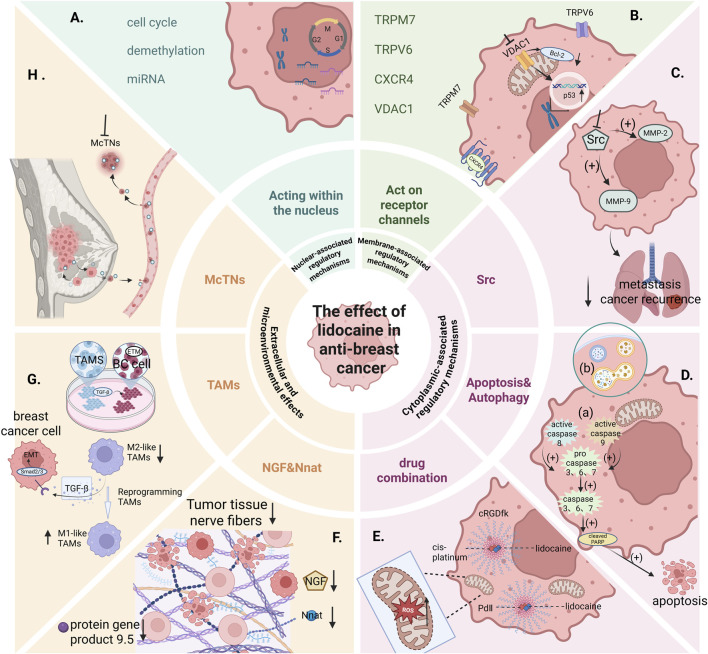
Lidocaine plays a crucial role in surgical procedures. Beyond its inherent analgesic effects, it also exhibits multiple inhibitory effects on breast cancer. Research has shown that lidocaine can regulate nuclear substances, exerting inhibitory effects **(A)**. By targeting related channels and receptors on the plasma membrane and mitochondrial membrane, lidocaine inhibits the viability and migration of breast cancer cells **(B)**. Lidocaine reduces lung metastasis by inhibiting Src, which in turn downregulates the expression of MMP-2 and MMP-9 **(C)**. Additionally, lidocaine promotes autophagosome-lysosome formation in breast cancer cells and is involved in caspase-dependent apoptosis signaling pathways **(D)**. When used in combination with other anti-breast cancer drugs, lidocaine enhances the therapeutic efficacy of each agent **(E)**. Lidocaine also downregulates the expression of protein gene product 9.5, neurofilaments, nerve growth factor, and neural components, thereby inhibiting the growth of breast cancer cells **(F)**. Co-culture with tumor-associated macrophages (TAMs) and triple-negative breast cancer (TNBC) cells reveals that lidocaine reduces M2-type TAMs, increases M1-type TAMs, and induces the reprogramming of M2 to M1. This process decreases TGF-β/Smads-mediated epithelial-mesenchymal transition (EMT) signaling, thereby inhibiting cancer metastasis and recurrence **(G)**. Lidocaine also reduces the adhesion of circulating breast cancer cells by inhibiting McTNs **(H)**. (Created with BioRender.com).

This section explores the key nuclear-associated regulatory mechanisms involved in lidocaine’s anticancer effects, including the functional modulation of Cdc20, DNA demethylation, and miRNA-mediated transcriptional regulation as depicted in Figure 1A.

### 2.1 Cdc20-centered nuclear regulation of cell cycle progression

Inducing mitotic arrest through inhibition of mitotic progression represents an effective strategy to suppress cancer cell proliferation.

**FIGURE 2:**
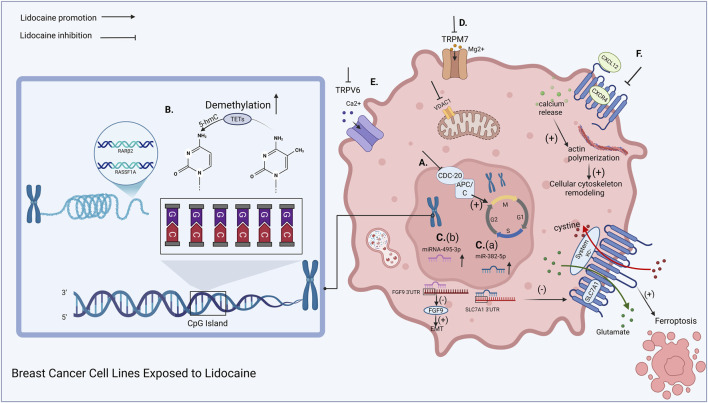
Breast cancer cell lines exposed to lidocaine. Lidocaine inhibits the key cell cycle protein CDC 20, causing breast cancer cells to arrest at the G2/M phase **(A)**. Lidocaine significantly increases the expression of specific miRNAs in breast cancer cells and is associated with suppressed cancer cell growth **(B)**. Lidocaine induces DNA demethylation in breast cancer cell lines **(C)**. Lidocaine inhibits the function of TRPM7 **(D)**. Lidocaine downregulates the expression of TRPV6, reducing the rate of Ca^2+^ influx **(E)**. Lidocaine suppresses CXCL12-induced CXCR4 signaling, impairing the fundamental signaling cascade of cytoskeletal remodeling **(F)**. (Created with BioRender.com).

Cell division cycle 20 (Cdc20) is a critical mitotic factor and a key partner in the anaphase-promoting complex/cyclosome (APC/C). Elevated levels of Cdc20 lead to cell cycle dysregulation by overriding the spindle assembly checkpoint (SAC), resulting in disrupted proliferation and tumorigenesis (Greil et al., 2022). Inhibiting Cdc20-APC/C activity or inducing protein degradation to eliminate Cdc20 has emerged as an effective strategy for cancer therapy, as depicted in Figure 2A.

Cdc20 expression is closely linked to poor prognosis in triple-negative breast cancer (TNBC), and targeted inhibition of Cdc20 could be an effective anti-mitotic therapeutic strategy (Chi et al., 2019). Lidocaine has been identified as a potential Cdc20 inhibitor with high drug-likeness. A study by Das et al. showed that lidocaine interacts with the active site of Cdc20, making its inhibitory effect on breast cancer cells competitive. Treatment with lidocaine downregulated the Cdc20 gene expression in TNBC cells (Das et al., 2023). Similarly, Zhao et al. demonstrated that CDC20 inhibition enhances radiosensitivity by suppressing proliferation, aggravating DNA damage, inducing G2/M phase arrest, and promoting apoptosis in hepatocellular carcinoma cells (Zhao et al., 2021).

The same group further explored the anti-tumor effects of lidocaine on MDA-MB-231 cells. Within 24 h of treatment, a sixfold increase in apoptotic cells was observed following lidocaine treatment, including both early and late apoptosis (Das et al., 2023). Another study reported the synergistic anti-tumor effects of lidocaine combined with the FDA-approved CDK4/6 inhibitor palbociclib in TNBC (Han et al., 2022). While CDK4/6 plays a role in the G1-S transition, Cdc20 is essential for the mid-to-late transition. Exploring the role of Cdc20 in the synergistic anti-cancer effect of lidocaine and palbociclib would be valuable.

Das et al. reported that lidocaine at a concentration of 467 μM significantly induced G2/M phase arrest in MDA-MB-231 cells and suggested that its anti-tumor activity in TNBC cells may be mediated, at least in part, through the induction of G2/M arrest and apoptosis. The study also found that lidocaine inhibited 50% of TNBC cell viability with minimal effects on non-cancerous breast epithelial cells (Das et al., 2023). This mechanism may be attributed to the identification of lidocaine as a potential Cdc20 inhibitor with high drug similarity (Das et al., 2023). Long et al. used flow cytometry to measure the impact of 0, 1, and 3 mM lidocaine on the cell cycle distribution of MCF-7 cells. The 3 mM group showed a significantly higher proportion of cells in the G2/M phase compared to the control group (Long et al., 2022).

Regarding cell cycle regulation, Li et al. demonstrated that treatment with six common local anesthetics at both plasma (∼10 μM) and 10 × plasma concentrations led to a significant increase in S-phase cells and a corresponding decrease in G0/G1-phase cells in MDA-MB-231 cells. This indicates that local anesthetics can stall the cell cycle during DNA replication, potentially leading to apoptotic arrest. Their quantitative analysis further revealed that while clinical plasma concentrations had minimal impact on cell viability, 10 × plasma concentrations for 72 h significantly inhibited cell viability; specifically, bupivacaine, levobupivacaine, and chloroprocaine reduced MDA-MB-231 viability by more than 40%, while levobupivacaine decreased MCF7 viability by 50%. Additionally, MDA-MB-231 cell migration was effectively suppressed by 10 × plasma concentrations of levobupivacaine, ropivacaine, and chloroprocaine. Importantly, these agents showed no cytotoxicity toward the non-cancerous breast cell line MCF 10A, highlighting a selective inhibitory effect on malignant cells that depends on the specific agent, concentration, and exposure time (Li et al., 2018).

Notably, accumulating evidence suggests that lidocaine-induced cell cycle arrest may not be exclusively mediated through Cdc20-dependent mitotic regulation, but can also involve alternative nuclear-associated pathways regulating earlier phases of the cell cycle.

In this context, a recent study focusing on HER2-positive breast cancer cell lines (AU565 and BT474) demonstrated that lidocaine significantly inhibited cell viability and proliferation by inducing G0/G1 cell cycle arrest. Mechanistically, lidocaine downregulated O-GlcNAc transferase (OGT), leading to a global reduction in protein O-GlcNAcylation. OGT was shown to interact with and stabilize cyclin L1 (CCNL1) via O-GlcNAcylation, a modification critical for CCNL1-mediated cell proliferation. Importantly, rescue experiments revealed that overexpression of either OGT or CCNL1 partially reversed the antiproliferative effects of lidocaine, supporting a causal role of the OGT–CCNL1 axis in lidocaine-induced growth suppression (Sun et al., 2025).

Together, these findings indicate that while Cdc20 represents a key nuclear regulator mediating lidocaine-induced mitotic arrest at the G2/M transition, lidocaine can also modulate breast cancer cell proliferation through Cdc20-independent nuclear mechanisms, particularly those governing G0/G1 phase progression in a subtype-specific manner.

### 2.2 DNA demethylation–associated transcriptional regulation

DNA methylation is one of the earliest and most widely studied epigenetic modifications, regulating various cellular processes, including transcription regulation, transposon silencing, maintenance of genomic imprinting, and X-chromosome inactivation (Baccarelli and Ordovás, 2023). Methylation-induced gene silencing involves complex interactions between DNA-protein and protein-protein interactions, as well as the activity of various enzymes responsible for dynamic changes in DNA methylation patterns. Key participants in this process include DNA methyltransferases (DNMTs) and Ten-eleven translocation enzymes (TETs), which coordinate the delicate balance of DNA methylation dynamics (Shi et al., 2021). Understanding the complex mechanisms of DNA methylation-mediated gene regulation is crucial for elucidating the pathogenesis of breast cancer and may pave the way for the development of targeted interventions.

Abnormal DNA methylation is a hallmark of cancer and is associated with aberrant gene expression (Cheishvili et al., 2015). Retinoic acid receptor β (RARβ2) and Ras association domain family 1A (RASSF1A) are considered effective tumor suppressor genes. Restoring RARβ2 expression in RARβ2-negative cancer cells can restore retinoic acid-induced growth inhibition and reduce tumorigenicity. Methylation of these tumor suppressor genes silences their expression and promotes tumorigenesis in breast cancer (Sirchia et al., 2002; Li et al., 2014).

Li et al. demonstrated that lidocaine promotes significant global genomic demethylation in human breast cancer cells, including MCF-7 and MDA-MB-231, specifically targeting the promoters of RARβ2 and RASSF1A, as depicted in Figure 2B. Crucially, the study found that lidocaine-mediated demethylation sensitized breast cancer cells to the cytotoxicity of cisplatin. Combined treatment with lidocaine and cisplatin promoted significantly higher levels of apoptosis in MCF-7 cells compared to treatment with either agent alone. Furthermore, the abrogation of RARβ2 or RASSF1A expression was shown to inhibit this enhanced apoptosis, proving that the demethylation of these specific genes is essential for lidocaine’s ability to potentiate cisplatin’s efficacy.

Supporting these findings, Lirk et al. demonstrated that lidocaine induces time- and dose-dependent DNA demethylation in the estrogen receptor (ER)–negative BT-20 breast cancer cell line at clinically relevant concentrations. In the ER-positive MCF-7 cell line, the demethylating effect was less pronounced but remained statistically significant, likely due to variations in baseline DNA methylation levels (Lirk et al., 2012). Subsequent studies by the same group further reported that both lidocaine and ropivacaine exerted demethylating effects in BT-20 and MCF-7 cells, whereas bupivacaine did not exhibit such activity. Moreover, lidocaine showed an additive demethylating effect when combined with the chemotherapeutic DNA methyltransferase inhibitor 5-aza-2′-deoxycytidine (DAC) (Lirk et al., 2014).

### 2.3 miRNA-mediated regulation associated with nuclear processes

Although miRNAs function in diverse cellular compartments, the canonical miRNA biogenesis pathway originates in the nucleus. On this basis, we include the present discussion under miRNA-mediated regulation associated with nuclear processes. MiRNAs are small non-coding RNAs that regulate gene expression by suppressing target mRNA translation through the RNA-induced silencing complex (RISC) (Lei et al., 2022).

#### 2.3.1 miR-382-5p/SLC7A11–associated regulatory axis

Ferroptosis is a novel form of cell death associated with the accumulation of lipid reactive oxygen species (ROS) induced by iron (Dixon et al., 2012). Increasing evidence suggests that targeting ferroptosis could be a viable strategy for treating breast cancer (Li et al., 2020). Studies have shown that TNBC patients are more sensitive to ferroptosis than estrogen receptor-positive patients, offering a potential therapeutic approach for TNBC patients (Doll et al., 2017).

Sun et al.’s research found that the expression of miR-382-5p in the T47D breast cancer cell line significantly increased with rising lidocaine concentrations, with the most pronounced effect at 3 mM. In a subcutaneous xenograft mouse model, the miR-382-5p expression in T47D breast cancer tissues was significantly higher in the lidocaine-treated group than in the control group. TargetScan prediction revealed that miR-382-5p has a potential binding site in the 3′UTR of SLC7A11. A luciferase reporter assay confirmed that miR-382-5p significantly inhibited the luciferase activity of the wild-type SLC7A11. Treatment with lidocaine significantly reduced the SLC7A11 protein levels in T47D breast cancer cells, while using an inhibitor to suppress miR-382-5p reversed this effect, indicating that miR-382-5p is a key mediator of lidocaine’s regulation of SLC7A11. Sun et al.'s study suggests that the miR-382-5p axis may be a potential therapeutic target for breast cancer treatment, as depicted in Figure 2C (a) (Sun et al., 2021).

The cystine/glutamate antiporter SLC7A11 (also known as xCT) is crucial in the ferroptosis process of breast cancer cells. SLC7A11 is overexpressed in various cancer cells to meet the high demand for antioxidant defense. Its function is to transport cystine for glutathione synthesis, playing a vital role in antioxidant defense (Koppula et al., 2021). Breast cancer cells primarily rely on the cystine transporter system x_c_^−^ to take up cystine, which is then converted to cysteine in the cytoplasm through NADPH-consuming reduction reactions. Cysteine is used for glutathione synthesis, and glutathione peroxidase 4 (GPX4) uses glutathione (GSH) to reduce lipid hydroperoxides (LOOH) into lipid alcohol (LOH), thereby inhibiting ferroptosis.

#### 2.3.2 miR-495-3p/FGF9–associated regulatory axis

Lin et al. demonstrated that lidocaine treatment significantly upregulated miR-495-3p expression in MCF-7 cells, leading to a marked inhibition of cell proliferation, migration, and invasion. Bioinformatic analysis and dual-luciferase reporter assays confirmed FGF9 as a direct target of miR-495-3p as depicted in Figure 2C (b). Specifically, lidocaine-induced miR-495-3p upregulation resulted in the downregulation of FGF9 protein, alongside reduced expression of its downstream effectors, including Cyclin D1 (a proliferation marker), as well as MMP-2 and MMP-9 (matrix metalloproteinases associated with invasion). Rescue experiments using FGF9 overexpression or miR-495-3p inhibitors significantly attenuated the inhibitory effects of lidocaine on these malignant phenotypes. These findings underscore that lidocaine exerts its anti-breast cancer effects by modulating the miR-495-3p/FGF9 axis, thereby disrupting the regulatory networks involved in epithelial–mesenchymal transition (EMT) and tumor invasiveness (Lin et al., 2021).

Although mechanistic studies of miR-495-3p have been more extensively explored in other malignancies, such as gastric cancer, where it has been shown to regulate autophagy-related pathways and drug resistance through targets including GRP78, these observations primarily serve to support a broader tumor-suppressive role of miR-495-3p (Chen et al., 2018; Eun et al., 2018). In the context of breast cancer, current evidence underscores the importance of the miR-495-3p/FGF9 axis as a lidocaine-responsive regulatory pathway.

## 3 Membrane-associated regulatory mechanisms

In addition to regulating cell proliferation and apoptosis through nuclear mechanisms, lidocaine also inhibits breast cancer cell viability and migration by targeting membrane-associated channels and receptors at both the plasma and mitochondrial membranes, as depicted in Figure 1B.

### 3.1 Cell membrane–associated regulatory mechanisms

#### 3.1.1 Lidocaine-associated functional modulation of TRPM7

Transient receptor potential melastatin 7 (TRPM7) is a channel permeable to Ca^2+^, Mg^2+^, and Zn^2+^ ions. It is abnormally expressed in several cancers and may be involved in disease progression (Gautier et al., 2016). The regulation of intracellular Ca^2+^ homeostasis by TRPM7 is believed to be associated with cancer development (Jiang et al., 2007). A study by Liu et al. investigated the effects of lidocaine on TRPM7 function, cell viability, and migration in human breast cancer cell lines expressing TRPM7. The results showed that lidocaine significantly reduced cell viability and migration of the tested breast cancer cell lines, including MDA-MB-231, AU565, T47D, and MDA-MB-468. These findings suggest that TRPM7 is a potential target mediating lidocaine’s effects on cell viability and migration (Liu H et al., 2021), as depicted in Figure 2D. Guilbert et al. also demonstrated that TRPM7 is essential for breast cancer cell proliferation (Guilbert et al., 2009). TRPM7 overexpression has been reported to promote bladder cancer cell proliferation (Gao et al., 2017), and pancreatic cancer cell migration is regulated by a Mg^2+^-dependent mechanism involving TRPM7 (Rybarczyk et al., 2012).

#### 3.1.2 Lidocaine-associated functional modulation of TRPV6

TRPV6 is a member of the TRP channel family characterized by high Ca^2+^ selectivity (Peters et al., 2012). Selective silencing of TRPV6 has been shown to inhibit migration and invasion in MDA-MB-231 and MCF-7 cells (Dhennin-Duthille et al., 2011). Lidocaine’s inhibitory effect on TRPV6-expressing cancer cells is associated with reduced calcium influx and downregulated TRPV6 expression, as depicted in Figure 2E. From a clinical perspective, these findings raise the possibility that local anesthetics could be beneficial in tumors characterized by elevated TRPV6 expression (Jiang et al., 2016). TRPV6 mRNA and protein expression has been detected in ovarian cancer and other cancer types, such as breast cancer, prostate cancer, thyroid cancer, and colorectal cancer (Bowen et al., 2013). Previous studies have shown that TRPV6 has diagnostic, prognostic, and therapeutic potential in human breast cancer and prostate cancer (Peng et al., 2001; Bolanz et al., 2009; Gkika and Prevarskaya, 2011). TRPV6 is highly expressed in estrogen receptor-negative breast cancer (Bowen et al., 2013), and therefore, lidocaine’s inhibitory effect on TRPV6 may be particularly beneficial for the ER-negative subtype.

#### 3.1.3 Lidocaine-associated functional modulation of CXCR4

The chemokine system serves as a key communication bridge between tumor cells and stromal cells, crucial for sustaining tumor growth and metastasis. The chemokine receptor CXCR4 and its ligand CXCL12 are widely expressed in various cancer types, making both targets for cancer therapy (Guo et al., 2016). Numerous studies have emphasized the critical role of the CXCL12/CXCR4 axis in the metastasis of breast cancer cells to the bone, lung, and brain (Chiang and Massagué, 2008).

D’Agostino et al. found that lidocaine inhibits CXCL12-induced CXCR4 signaling, disrupting the cytoskeletal remodeling necessary for breast cancer cell migration (D’Agostino et al., 2018), as depicted in Figure 2F. This suggests that lidocaine may be a potential therapeutic approach to inhibit breast cancer recurrence and metastasis. At clinically relevant concentrations, lidocaine inhibits CXCR4-mediated cell migration by targeting its downstream signaling cascades. Lidocaine impairs directed cell migration by inhibiting CXCL12-dependent calcium release and actin polymerization, essential for cytoskeletal remodeling. Notably, high doses of lidocaine induce the loss of cortical actin stress fibers, indicating a change in the basal cytoskeletal structure (D’Agostino et al., 2018).

Growing evidence suggests that different types of anesthetics may have pro-metastatic or anti-metastatic effects, depending on the cancer cell type, dosage, and administration regimen (Yang et al., 2017). For instance, volatile anesthetics have been described to potentially promote metastasis via CXCR2 expression in ovarian cancer cells (Iwasaki et al., 2016).

### 3.2 Mitochondrial membrane receptor -associated regulatory mechanisms

#### 3.2.1 Lidocaine-associated functional modulation of VDAC1

Mitochondrial bioenergetics, biosynthesis, and signaling are closely linked to tumorigenesis. Voltage-dependent anion channels (VDAC), located on the outer mitochondrial membrane, are pore-forming ion channel proteins. VDAC1, one of the most studied members of the VDAC family, acts as a gatekeeper of the mitochondria, controlling the exchange of metabolites, fatty acids, ions, calcium, reactive oxygen species, and cholesterol between the cytoplasm and mitochondria. VDAC1, in complex with the glycolytic hexokinase, favors the preferential uptake of ATP produced by mitochondria to maintain the high glycolytic flux of tumors, making VDAC1 a potential target for anti-cancer therapy (Krasnov et al., 2013).

The expression of the VDAC1 gene in breast cancer tissues is significantly higher than in normal breast tissues from the same patients. Research by Long et al. showed that lidocaine significantly inhibited the expression of VDAC1 in MCF-7 cells. Additionally, the expression of VDAC1 and the cell death-inhibiting protein Bcl-2 in MCF-7 cells decreased markedly, while the tumor suppressor gene p53 expression increased significantly (Long et al., 2022), as depicted in Figure 1B.

## 4 Cytoplasmic-associated regulatory mechanisms

Beyond nuclear-associated regulation, lidocaine also exerts anticancer effects through cytoplasmic-associated regulatory events. These include the modulation of Src-related cytoplasmic activity, the involvement of caspase-dependent apoptotic signaling and autophagy-related processes, as well as alterations in intracellular responses observed under combination treatment with other anticancer agents.

### 4.1 Lidocaine-associated SRC-related cytoplasmic signaling modulation

Johnson et al. administered intravenous lidocaine (1.5 mg/kg bolus, followed by a 25-minute infusion of 2 mg/kg) to 4T1 breast cancer mice perioperatively, and found that lidocaine reduced lung metastasis colony counts compared to sevoflurane treatment alone (Johnson et al., 2018). Later, Wall et al. conducted *in vitro* studies showing that lidocaine may prevent metastasis by acting on the tyrosine kinase Src pathway. They found that after the death of mice bearing 4T1 breast cancer cells, the serum levels of Src pathway-associated metastasis enzymes MMP-2 and MMP-9 were decreased (Wall T. P. et al., 2019), as depicted in Figure 1C. At the same time, Freeman et al. reported in their study involving female BALB/c mice bearing 4T1 cells that lidocaine and propofol alone reduced lung metastasis colony counts 2 weeks post-surgery, whereas methylprednisolone increased lung metastasis (Freeman et al., 2019).

Subsequent studies by Wall et al. further demonstrated that lidocaine and bosutinib, either alone or in combination, inhibited 4T1 cell viability and migration, but this effect was only evident at supra-therapeutic concentrations. While lidocaine may suppress 4T1 metastasis *in vivo*, it did not have a direct effect on 4T1 cells at non-toxic concentrations. The anti-metastatic properties of lidocaine may be complex and multifactorial, and challenging to replicate outside of biological hosts (Wall et al., 2021).

Src family non-receptor tyrosine kinases regulate various cellular processes, including proliferation, differentiation, migration, cellular viability, and angiogenesis. Src is often abnormally activated in solid tumors, promoting tumor progression and metastasis (Shah and Gallick, 2007). Overexpression of Src is associated with the progression of various human cancers, including breast, colon, and pancreatic cancers (Johnson and Gallick, 2007). Src activation increases the expression of matrix metalloproteinases (MMPs), including MMP-2 and MMP-9, which degrade the extracellular matrix and promote cancer cell migration and invasion (Roy et al., 2009).

### 4.2 Caspase-dependent apoptotic signaling and autophagy-related processes

#### 4.2.1 Lidocaine-associated caspase-dependent apoptotic signaling

Several studies have reported that lidocaine promotes caspase-dependent apoptosis in breast cancer cells, as evidenced by increased caspase-3/7 activation and PARP cleavage, suggesting functional crosstalk between autophagy and apoptotic signaling.

A study by Chang et al. found that lidocaine and bupivacaine treatment induced apoptosis and suppressed cell viability in MCF-7 breast cancer cells (Chang et al., 2014). Lidocaine treatment induced the cleavage of caspase 7, 8, and 9 and poly (ADP-ribose) polymerase (PARP), as depicted in Figure 1D (a). The cleavage of caspase 7 and PARP induced by local anesthetics was effectively blocked by caspase inhibitors. Additionally, treatment of MCF-7 xenografts with local anesthetics resulted in increased caspase 7 cleavage and increased terminal deoxynucleotidyl transferase-mediated dUTP nick end labeling (TUNEL) staining.

#### 4.2.2 Lidocaine-associated autophagy-related regulatory processes

Autophagy is a lysosome-dependent degradative process involved in the turnover of intracellular components and damaged organelles, and has been implicated in regulating cell fate under stress conditions (Mulcahy Levy et al., 2017). Functionally, autophagy proceeds through a coordinated sequence of membrane remodeling events culminating in lysosomal degradation. Depending on the cellular context, autophagy has been reported to contribute to cell death through distinct modes, including autophagy-associated, autophagy-mediated, or autophagy-dependent mechanisms. However, the specific form of autophagy responsible for lidocaine-induced breast cancer cell death has not yet been clearly defined.

Chen et al. observed that lidocaine, levobupivacaine, bupivacaine, and ropivacaine exhibited cytotoxicity against two breast cancer cell lines, MDA-MB-231 and BT-474. In addition to inducing apoptosis and inhibiting cell proliferation, the four local anesthetics reduced ROS levels and increased autophagy biomarkers (i.e., LC3B II/I ratio). Among them, lidocaine significantly promoted the formation of autophagic lysosomes in MDA-MB-231 cells in a dose-dependent manner, as depicted in Figure 1D (b), but not in BT-474 cells (Chen et al., 2022). Recent studies have increasingly shown a connection between autophagy and cancer (Dower et al., 2018).

### 4.3 Lidocaine-centered cytoplasmic responses under combination or co-delivery strategies

The side effects of chemotherapy can reduce the quality of life for cancer patients. One limitation of using chemotherapy agents alone is their inability to inhibit tumor metastasis, and increasing the dose can lead to more severe adverse reactions. Combination therapies provide limitless possibilities for improving chemotherapy effectiveness.

#### 4.3.1 Lidocaine-mediated sensitization to palbociclib

Lidocaine can act as a chemotherapeutic sensitizer and alleviate pain in certain cancers. Palbociclib is a potent cyclin-dependent kinase (CDK) 4/6 inhibitor approved for the chemotherapy of advanced breast cancer. Research by Han et al. demonstrated that, in an orthotopic breast cancer model, combination treatment with lidocaine and palbociclib significantly inhibited tumor growth and increased tumor cell apoptosis compared to monotherapy. By inhibiting the PI3K/AKT/GSK3β pathway, the combination of palbociclib and lidocaine exhibited a synergistic anti-cancer effect in breast cancer cells, suggesting that this combination may be an effective therapeutic approach for breast cancer (Han et al., 2022).

#### 4.3.2 Nanocarrier-mediated targeted delivery of lidocaine in breast cancer cells

A study by Gao et al. developed a tumor-targeted dual-drug nanogel system co-loaded with lidocaine and cisplatin using cRGDfk-modified nanogels. The introduction of lidocaine not only promoted cisplatin-induced apoptosis both *in vitro* and *in vivo* but also alleviated the metastasis of MDA-MB-231 breast cancer cells in a mouse model. Additionally, cisplatin-induced weight loss was mitigated, allowing for higher dose efficacy with less weight loss, indicating that the adverse effects of cisplatin chemotherapy were reduced. Furthermore, the introduction of the peptide fragment cRGDfk, which has a high affinity for the αvβ3 integrin, further enhanced the accumulation of the drug-loaded nanogels at the tumor site, inhibiting primary tumor growth. These results suggest that ligand-modified nanogels co-loaded with lidocaine and cisplatin represent a promising strategy for the combined treatment of breast cancer with high αvβ3 integrin expression, as depicted in Figure 1E (Gao et al., 2018).

Similarly, zeolitic imidazolate framework-8 (ZIF-8) nanoparticles have been developed to selectively deliver lidocaine to breast cancer cells, improving cellular uptake and enhancing anticancer effects (di Nicola et al., 2025). These studies collectively demonstrate that nanocarrier-mediated delivery strategies can potentiate the cytotoxicity of lidocaine-based therapies while minimizing off-target effects.

#### 4.3.3 Anticancer effects of lidocaine–palladium (II) complexes

The clinical success of anticancer drugs such as cisplatin, carboplatin, and oxaliplatin has prompted researchers to further develop novel metal-based anticancer drugs to treat cancer, reduce toxic side effects, and overcome platinum resistance. Divalent palladium complexes containing phenylacrylamide ligands may form bioactive compounds with mechanisms of action similar to cisplatin, and due to their lipophilic properties, they hold significant potential as anticancer drugs. The lidocaine and phenylacrylamide palladium (II) complexes 1-3 demonstrate notable *in vitro* antiproliferative activity against human ovarian cancer (A2780), colorectal cancer (HT29), breast cancer (MCF-7), hepatocellular carcinoma (HepG-2), and lung adenocarcinoma (A549) cells (Tabrizi and Chiniforoshan, 2016).

Mechanistic studies show that the cytotoxicity of the Pd(II) complexes is primarily associated with their accumulation in cancer cells and the increase in intracellular reactive oxygen species (ROS) levels, leading to the loss of mitochondrial membrane potential and the induction of apoptosis, as depicted in Figure 1E. Oxidative stress is caused by an imbalance between ROS production and antioxidant capacity (Sies et al., 2017). Many studies have indicated that elevated oxidative stress is a potential strategy for anticancer treatment (Gupte and Mumper, 2009; Nicco and Batteux, 2017). These complexes increase ROS production in the cellular environment, leading to the loss of mitochondrial membrane potential and ultimately resulting in cancer cell death. This provides new insights for the development of tumor-selective palladium (II) complex-based anticancer drugs.

## 5 Extracellular and microenvironmental effects

Apart from intracellular regulatory mechanisms, lidocaine has also been shown to influence breast cancer progression through extracellular and tumor microenvironment–related effects.

### 5.1 Lidocaine-mediated suppression of neurofiber formation associated with reduced NGF and Nnat expression in the tumor microenvironment

A study by Li et al. explored the relationship between lidocaine and the circulation of breast cancer cells through the perspective of neurofiber formation. In mice subcutaneously implanted with MDA-MB-231 and 4T1 cell lines to simulate tumor self-seeding caused by circulating cancer cells, lidocaine treatment effectively inhibited tumor growth and neurofiber formation. Silver staining was used to observe the distribution of neurofibers in tumor tissues, and immunohistochemistry was performed to assess the expression levels of neuro-related proteins. Lidocaine treatment significantly downregulated the expression levels of protein gene product 9.5, neurofilament, nerve growth factor (NGF), and neural elements (Nnat). Overexpression of NGF and Nnat reversed the therapeutic effects of lidocaine. These findings suggest that lidocaine inhibits neurofiber formation by targeting Nnat and regulating NGF production in cancer cells, potentially suppressing breast cancer invasion and metastasis (Li et al., 2021), as depicted in Figure 1F.

### 5.2 Lidocaine-mediated regulation of tumor-associated macrophage (TAM) polarization

Macrophages play a key role in cancer growth and metastasis (Zhang et al., 2012; Liu J et al., 2021). M2 polarization of macrophages is involved in cancer metastasis, and anesthetics are associated with macrophage polarization (Qian and Pollard, 2010; Yu and Bai, 2022; Zhao and Yun, 2022). Therefore, the presence of tumor-associated macrophages (TAMs) in the tumor microenvironment is often linked to poor prognosis and increased metastatic potential (Lin et al., 2019). Moreover, M2-TAMs secrete cytokines and growth factors that promote cancer cell growth, migration, and invasion. Previous studies have shown that TGF-β secreted by M2 macrophages promotes glioma cell stemness and migration through the Smad2/3 signaling pathway (Fabregat et al., 2016).

Research by Seok Han et al. found that lidocaine, when combined with propofol or sevoflurane, inhibited the growth of TNBC cells. This combination treatment effectively suppressed cancer cell migration and invasion. In an orthotopic breast cancer model, lidocaine inhibited tumor growth and prolonged overall survival. When TAMs were co-cultured with TNBC cells, lidocaine not only reduced the increase in M2-type TAMs caused by sevoflurane or propofol, but also increased M1 macrophage polarization, thereby hindering TNBC tumor growth. Additionally, they discovered that TGF-β secreted by TAMs increased EMT signaling in TNBC cells, and lidocaine not only acted on cancer cells but also reprogrammed M2-TAMs into M1-TAMs. This reprogramming decreased TGF-β/Smad-mediated EMT signaling in TNBC cells, thereby inhibiting cancer metastasis and recurrence (Seok Han et al., 2024), as depicted in Figure 1G. These findings suggest that lidocaine, in combination with general anesthetics, may provide a potential therapeutic approach to inhibit recurrence and metastasis in breast cancer patients undergoing radical resection.

M2-like macrophages are immune cells found in the tumor microenvironment (TME). They are derived from circulating monocytes recruited to the tumor site, where they differentiate into tumor-associated macrophages and become a major component of the TME. In certain cases, M2-TAMs promote tumor growth and metastasis by secreting factors that stimulate angiogenesis, suppress immune responses, and remodel the extracellular matrix. Additionally, during surgery, cancer cells stimulate M2-TAM activity, leading to increased tumor growth and metastasis. M2-TAMs also secrete cytokines and growth factors that activate EMT signaling pathways, enabling cancer cells to acquire invasive and migratory characteristics to induce metastasis. Thus, the activation and polarization of TAMs in the TME have been identified as potential therapeutic targets. However, the impact of anesthetics on M2-TAMs in the cancer microenvironment across different tumor types remains to be further elucidated.

### 5.3 Lidocaine-mediated effects on Tubulin microtentacles (McTNs)

Metastatic dissemination involves multiple sequential steps, beginning with tumor cell detachment from the primary lesion, followed by survival in circulation and reattachment at distant sites. Detached breast cancer cells have been shown to form dynamic microtubule-based protrusions termed microtentacles (McTNs), which are mechanistically distinct from actin-based filopodia or invadopodia and facilitate efficient tumor cell reattachment (Yoon et al., 2011), as depicted in Figure 1H.

In this context, Yoon et al. investigated the effects of the local anesthetics lidocaine and tetracaine, which function as kinesin motor protein inhibitors, on McTN formation and function in both non-tumorigenic mammary epithelial cells and breast cancer cells. Treatment with either anesthetic induced rapid collapse of vimentin filaments and disruption of McTN architecture. Notably, tetracaine markedly reduced intracellular kinesin motility, leading to centripetal retraction of McTNs and impaired tumor cell aggregation and reattachment, whereas lidocaine exerted similar but comparatively weaker effects. These findings suggest that lidocaine may attenuate metastatic potential, at least in part, through modulation of microtubule–vimentin dynamics mediated by kinesin inhibition.

Consistent with the functional importance of microtubule dynamics in metastatic seeding, *in vivo* studies in other cancer models have demonstrated that microtubule polymerization is critical for early tumor cell adhesion to the microvasculature, whereas actin polymerization may exert context-dependent effects on tumor cell attachment (Korb et al., 2004).

## 6 Clinical applications and Progress of lidocaine in cancer treatment

Surgical resection of the tumor is a primary treatment method for breast cancer and many other cancers (Martinez et al., 2015). In breast cancer surgery, the use of paravertebral anesthesia and analgesia can reduce the risk of recurrence or metastasis during the early follow-up period (Exadaktylos et al., 2006). Patients who received intravenous lidocaine during pancreatic cancer resection showed improved overall survival rates at 1 and 3 years (Zhang et al., 2020). Although there is insufficient preclinical evidence to demonstrate that clinically applicable doses can improve oncological outcomes, the ongoing research into intravenous lidocaine infusion holds promise for improving the prognosis of cancer surgery patients.

In a multicenter trial, Badweet al. conducted an open-label, multicenter, randomized trial to test the impact of preoperative local anesthetic infiltration around breast cancer on disease-free survival (DFS). The results showed that patients receiving lidocaine infiltration had improved 5-year DFS and overall survival rates, with relative risks reduced by 26% and 29%, respectively. Local recurrence and distant metastasis were significantly reduced (Badwe et al., 2023). In this trial, the tumor resection plane was located outside the infiltrated tissue, suggesting that the survival benefits provided by lidocaine in breast cancer patients may be mediated by mechanisms other than analgesia. In Badwe et al.’s study, distant metastasis in the TNBC subtype was more significantly reduced in patients receiving lidocaine infiltration, which may be related to lidocaine’s stronger inhibitory effects on Cdc20 and downregulation of TRPM7 in TNBC cells.

Importantly, the clinical observation that peritumoral lidocaine infiltration prior to tumor excision is associated with improved survival in breast cancer has prompted further investigation into the potential role of tumor innervation and neural activity in shaping tumor biology. In this context, activity-regulated cytoskeleton-associated protein (ARC), a gene whose expression is regulated by neuronal activity, has been proposed as a surrogate marker of tumor-associated neural signaling. Analyses of large breast cancer cohorts, including the SCAN-B and TCGA datasets, revealed that high ARC expression was significantly associated with reduced cancer cell proliferation, lower histological grade, decreased genomic instability, and improved disease-specific and overall survival, specifically in estrogen receptor–positive/human epidermal growth factor receptor 2–negative (ER+/HER2−) breast cancers. Furthermore, tumors with high ARC expression exhibited increased infiltration of stromal components and multiple immune cell populations, indicating a distinct tumor microenvironment characterized by restrained tumor proliferation and enhanced stromal–immune interactions. These associations were not observed in TNBC, highlighting subtype-specific differences in neural–microenvironmental regulation (Yee et al., 2025).

Although ARC itself is not a direct molecular target of lidocaine, these findings provide clinically relevant support for the hypothesis that lidocaine-mediated modulation of tumor-associated neural activity and the tumor microenvironment may contribute to improved oncological outcomes following breast cancer surgery.

The clinical intravenous infusion of lidocaine achieves blood concentrations of approximately 10–50 μM, which is consistent with the effective concentrations in basic research that inhibit CXCR4 and TRPM7. This validates the rationale for translating basic mechanisms to clinical concentrations.

## 7 Discussion and future perspectives

Lidocaine has been shown to reduce the cell viability of normal breast epithelial cells and various breast cancer cell lines, inhibiting tumor cell migration and impairing breast cancer cells’ ability to grow in an anchorage-independent manner. Intraperitoneal administration of lidocaine at 100 mg/kg in mice—corresponding to approximately 8 mg/kg in humans based on body surface area conversion—significantly prolonged overall survival in a peritoneal MDA-MB-231 tumor model (Reagan-Shaw et al., 2008; Chamaraux-Tran et al., 2018).

While lidocaine, as a local anesthetic, has already achieved significant clinical effects in relieving postoperative pain, reducing opioid use, and mitigating stress responses, recent studies suggest that it also holds potential in breast cancer treatment. Lidocaine inhibits breast cancer cell proliferation, migration, and metastasis through multiple mechanisms. However, despite increasing attention to its potential anticancer effects, several limitations remain in current research. First, the effective concentration range of lidocaine spans from micromolar to millimolar levels, which may result in significant variations in its effects across different experimental settings, yet this phenomenon has not been adequately explored in existing literature. Second, there is a discrepancy between *in vitro* and *in vivo* effects: *in vitro* experiments require relatively high concentrations of lidocaine (e.g., 3 mM to induce G2/M arrest in MCF-7 cells (Long et al., 2022), or 467 μM to induce apoptosis in MDA-MB-231 cells (Das et al., 2023)) to directly inhibit breast cancer cell viability; however, *in vivo* studies show that clinically relevant doses (e.g., 1.5 mg/kg bolus+2 mg/kg infusion (Wall T. P. et al., 2019)) can modulate the polarization of tumor-associated macrophages (TAMs) (Seok Han et al., 2024), inhibit neurofiber formation (Li et al., 2021), and reduce lung metastasis in 4T1 breast cancer. The relative contribution of the “high-concentration direct effect *in vitro*” versus the “low-concentration microenvironmental regulatory effect *in vivo*” remains to be elucidated and should be further investigated using “cell-animal co-culture models”. This discrepancy highlights a major translational challenge, as the antitumor concentrations commonly used *in vitro* far exceed those that can be safely achieved in clinical practice. Specifically, the “pharmacological disconnect” between these preclinical concentrations and clinical reality must be addressed. While *in vitro* models often employ lidocaine in the 1–5 mM range to demonstrate direct anti-tumor signaling, human plasma concentrations exceeding 21.3 μM (5 μg/mL) are strictly associated with severe Local Anesthetic Systemic Toxicity (LAST), which can lead to life-threatening CNS and cardiovascular complications (Neal et al., 2018). At plasma levels above approximately 5–8 μg/mL, early central nervous system toxicity may manifest as perioral numbness, tinnitus, dizziness, or seizures, whereas higher concentrations can precipitate cardiac arrhythmias, myocardial depression, and hemodynamic instability. This 50- to 100-fold discrepancy suggests that the direct cytotoxic mechanisms observed in laboratories are pharmacologically unattainable through systemic administration in patients. Therefore, the antitumor effects observed at clinically relevant doses are more likely to reflect indirect modulation of the tumor microenvironment rather than direct tumor cell killing.

Additionally, the variability in lidocaine sensitivity across different cell lines, as shown in Table 2, remains an underexplored topic, and the biological mechanisms behind these differences have not been sufficiently analyzed. Moreover, the diversity of lidocaine’s targets, the heterogeneity of breast cancer immunohistological types, individual variations in patients’ immune responses, and the influence of different surgical techniques make it more complex to delineate the specific role of lidocaine in perioperative cancer progression.

**TABLE 2:** Breast cancer subtypes classified by ER, PR, HER2, and Ki-67 expression.

Subtype	Cell line	Origin	Research purpose	Sensitivity data
Luminal A	T47D	Human (ER+, PR+)	Hormone receptor response (Yu et al., 2017)	1–3 mM (24 h): Viability↓(Liu H et al., 2021)
	MCF-7	Human (ER+, PR+)	Treatment response (Moon et al., 2020)	≥1 mM (4–24 h), the IC50 = 4.5 ± 0.26 mM (Chang et al., 2014)
Luminal B	BT-474	Human (ER+, PR+, HER2+)	HER2-targeted therapy (Lasfargues et al., 1979)	1–3 mM (24 h): Viability↓(Liu H et al., 2021)
HER2(+)	AU565	Human (HER2+, HER3+, HER4+)	HER2 mechanisms (Schrörs, 2020)	0.3 mM (24 h): Early viability↓(Liu H et al., 2021)
Basal like	MDA-MB-231	Human (TNBC)	Metastasis mechanisms (Tseng et al., 2017)	1–10 mM (4 h) Dose-dependent ↓(Jiang et al., 2016)
	MDA-MB-468	Human (TNBC; EGFR+)	Chemoresistance (Tseng et al., 2017)	1–3 mM (24 h), Viability ↓ (Liu H et al., 2021)
	4T1	Mouse (TNBC-like)	*In vivo* metastasis & immunity (Schrörs, 2020)	1.5–2 mg/kg/h, Reduced lung burden (Freeman et al., 2019)

Abbreviations: ER, Estrogen Receptor; PR, Progesterone Receptor; HER2, Human Epidermal Growth Factor Receptor 2; TNBC, Triple-Negative Breast Cancer; IC 50, half-maximal inhibitory concentration.

From a translational medicine perspective, the potential of lidocaine in cancer therapy still needs to be validated through more comprehensive preclinical and clinical trials. The effects of anesthetics on cancer treatment may be influenced by several variables, such as the type of anesthetic, tissue concentration, exposure time, and administration methods. Therefore, translational research should focus on the smooth transition from basic research to clinical practice, exploring lidocaine’s role at different stages of cancer treatment, especially its synergistic effects with other anticancer drugs. While preclinical studies provide evidence for lidocaine’s potential in anticancer therapy, clinical trials have yet to fully validate its widespread use in cancer treatment. The conflicting results in clinical trials may stem from overlooked confounding variables, such as the specific anesthetic technique employed. For instance, the use of Total Intravenous Anesthesia (TIVA) with lidocaine versus volatile anesthesia may lead to divergent oncological outcomes, as volatile agents themselves might influence the tumor microenvironment (Wall T et al., 2019). Future clinical studies must meticulously control for the timing of lidocaine administration to clarify its true therapeutic window. Future research should focus on the synergistic effects of lidocaine with existing treatment methods, the identification of optimal application protocols, and the development of personalized treatment strategies. The widespread clinical application of lidocaine in oncology will ultimately rely on multidisciplinary collaboration and the accumulation of robust long-term clinical evidence.
